# In vitro dissolution testing models of ocular implants for posterior segment drug delivery

**DOI:** 10.1007/s13346-021-01043-z

**Published:** 2021-08-11

**Authors:** Muhammad Faris Adrianto, Febri Annuryanti, Clive G. Wilson, Ravi Sheshala, Raghu Raj Singh Thakur

**Affiliations:** 1 School of Pharmacy, Medical Biology Centre, Queen’s University Belfast, 97 Lisburn Road, Belfast, BT9 7BL UK; 2 Department of Pharmaceutical Chemistry, Faculty of Pharmacy, Universitas Airlangga, Surabaya, East Java 60115 Indonesia; 3 Strathclyde Institute of Pharmacy and Biomedical Sciences, University of Strathclyde, 161 Cathedral Street, Glasgow, G4 0RE Scotland; 4 Department of Pharmaceutics, Faculty of Pharmacy, Universiti Teknologi MARA Selangor, Puncak Alam Campus, 42300 Bandar Puncak Alam, Kuala Selangor, Malaysia

**Keywords:** In vitro, Dissolution studies, Ocular implants, Posterior segment, Drug delivery

## Abstract

The delivery of drugs to the posterior segment of the eye remains a tremendously difficult task. Prolonged treatment in conventional intravitreal therapy requires injections that are administered frequently due to the rapid clearance of the drug molecules. As an alternative, intraocular implants can offer drug release for long-term therapy. However, one of the several challenges in developing intraocular implants is selecting an appropriate in vitro dissolution testing model. In order to determine the efficacy of ocular implants in drug release, multiple in vitro test models were emerging. While these in vitro models may be used to analyse drug release profiles, the findings may not predict in vivo retinal drug exposure as this is influenced by metabolic and physiological factors. This review considers various types of in vitro test methods used to test drug release of ocular implants. Importantly, it discusses the challenges and factors that must be considered in the development and testing of the implants in an in vitro setup.

**Graphical abstract:**

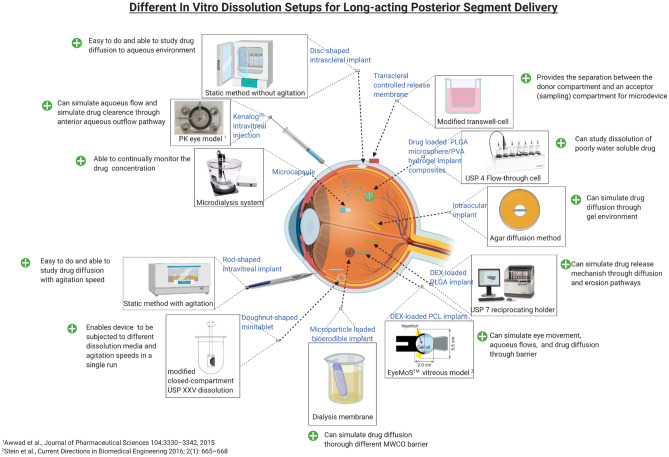

## Introduction

An appropriate in vitro setup is a primary quality control factor in pharmaceutical development for screening and selecting a suitable formulation. The design must be robust and be capable of a level of precision to assess the release of the formulation, test batch-to-batch reproducibility, the impact of processing changes and excipient-drug interactions.

In vitro dissolution testing becomes necessary to establish in vitro–in vivo correlations to allow pharmaceutical companies to apply for clinical test waivers, speeding up the transition from the laboratory to market and reducing the costs of medicines. In oral formulations, it led to the development of a multicompartmental apparatus to simulate different sections of the gut where the sequential changes in concentrations of surfactant and pH conditions could be shown to differentiate release between formulations [1]. This understanding was founded on physiology and anatomy, explicitly reproducing the agitation forces, composition and volume of gut liquids to see how they impacted the dissolution of the dosage form. This has been partially coupled with data that describes the absorption process. It remains a work in progress, with imperfect simulators that manage to capture some key attributes that drive drug release from complex formulations. Most importantly, it is based on continued exploration of which life processes can be ‘bottled’ or at least simulated in vitro with acceptable precision.

For any strategy in developing an in vitro test, the subtleties of organ structure and function must be understood, which is where this article starts. Unlike the gut, the eye is a closed system and the target neuronal tissue deep within protective connective tissue. The cornea is a surface structure, and so topical treatment might seem obvious: the problem reverts to a variant of dermal dosing. Unfortunately, this does not work because even disruption of the outermost water layer causes issues. This very organ is designed to resist investigation.

### The eye

The eye is the key sensory organ within the human body as it facilitates vision. It is a spherical object with an anterior–posterior diameter of around 22–27 mm and a circumference of 69–85 mm [2]. The globe’s anterior segment is comprised of the cornea, pupil, lens, iris, ciliary body, anterior chamber’s conjunctiva, trabecular meshwork and aqueous humour [3, 4]. The posterior segment contains the vitreous humour, a gel-filled sac behind the lens which extends to the macula and is sealed by the inner limiting membrane (ILM). The outer layers enclose the vitreous body and consist of connective, vascular and neural tissue: the sclera, choroid and most importantly, the retina. The external sclera contains blood vessels and provides attachments to the orbital muscles through the tendons, allowing the eye to make tracking movements.

The retina is a complex tissue packed with photoreceptors and interconnected through neurons to resolve the image projected through the cornea and lens to the retinal surface. The retina is a complex tissue filled with photoreceptors and interconnected through neurons to resolve the image projected through the cornea and lens to the retinal surface. There are around 91 million rod receptors and approximately 4.5 million cone receptors in the eye [5].Vision in poor light is provided by the rod-shaped photoreceptors widely dispersed at high density throughout the retina except in the central region at the posterior pole, where the density dramatically decreases. The macula is packed with cone receptors and provides sharp colour vision in good light. It is in line with the visual focus of the lens-retina axis. On examination, it appears as a darker portion of the retina, offset from the bright and noticeable optic disc. Information from the photoreceptors is relayed to the brain through the ganglions that gather at the optic disc and exit exteriorly the optic nerve. There are no photoreceptors on the surface of the optic disc, and this gives rise to the “blind spot” over the optic nerve head. Although the eye is likened to a camera, it has superiorities as revealed in a fascinating article by Roger Cicala [6]. The features of the human imaging system include the curvature of the retina and resolving power of the central retina, equivalent to 150 K pixels mm^−1^, an f-stop between 3.2 and 3.5 and a central visual angle of 55°. The total field of view is 160°, but much of the information from the 130 million receptors are lost as the optic nerve has only 1.2 million fibres. Each time a retinal receptor fires (depolarises), it must be brought back to ‘ready’. The efficiency of the system may only be 10% in real-time, so the eye scans laterally to maintain focus on moving objects. The brain stitches together the images to resolve detail, and sudden changes in movement are noted and may prompt a startle reaction eliciting a blink reflex. The reflex is not present at birth but may be produced in patients in a coma, particularly with a bright LED [7]. Voluntary avoidance movements are initiated on processing the signals. These features are essential because if vision is suddenly degraded by topical application, or a threat of moving to the eye at close range is detected, compliance is worsened by avoidance behaviour.

Ocular delivery is generally categorised into three types: topical delivery — includes lids, tear film and conjunctivae; periocular delivery — includes peribulbar, retrobulbar, subtenon and subconjunctival; and posterior delivery — includes vitreous humour, retina, choroid and macula. Systemic therapy is usually unsuccessful due to bystander effects and the barriers to drug delivery which impose structural, biochemical and physiological limitations as the target moves from surface to posterior pole. In behavioural reflexes, interfere with dosing including an inclination to turn the head away from a dispensing device.

**Fig. 1:**
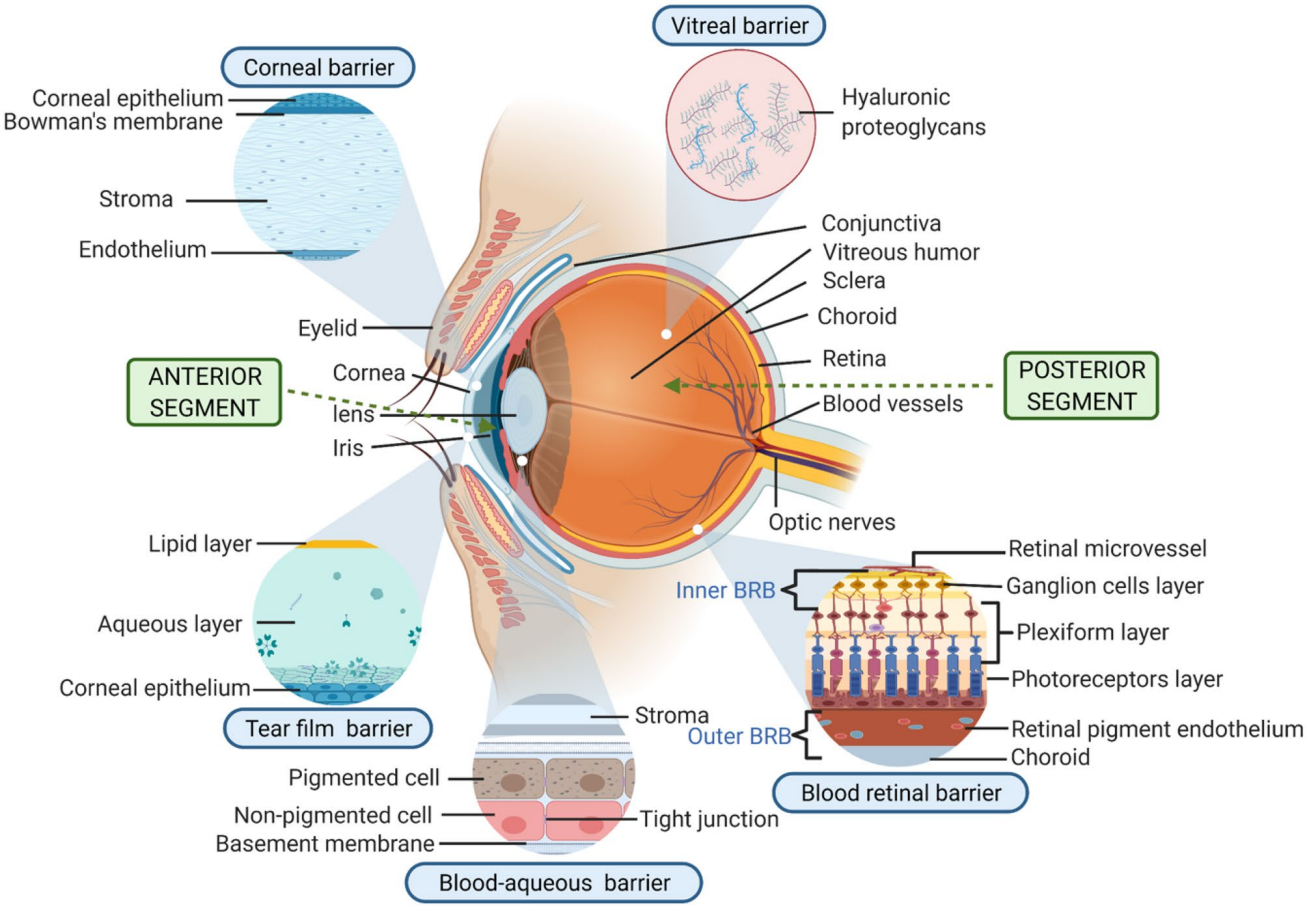
Physiological barriers in ocular drug delivery (created with BioRender.com)

The ocular tissue barriers are illustrated in Fig. 1, which are divided by location and nature. The barriers are not entirely static in nature as flows of liquid provide efficient clearance mechanisms such as blinking, increased lacrimation and outflow over the cheek, producing rapid elimination of topically applied drugs from the surface of the eye [8]. Within the eye, physical activity will exert convective forces as blinking compresses the cornea, pushing back the lens and causing minor distortions to the globe. The eyes scan from side to side by ballistic micromovements known as saccades to abruptly change the point of focus. The frequency and occurrence of saccadic tracking movements vary between species and may be partially responsible for differences seen in movement, erosion and dispersion of formulations in different laboratory animals. Additionally, the internal advective flows increase in old age as the interior vitreous becomes liquefied (syneresis), limiting drug effectiveness even when administered by the direct intravitreal route.

## Barrier for drug delivery to the posterior segment of the eye

The main goals of a drug delivery system include controlling the availability of the drug at the target site and maintaining the therapeutic levels for long-term treatment [9]. The ease of formulation, administration and safety/tolerability to the ocular tissues eye tissues must be addressed when designing a drug delivery system for the eye [10]. Four approaches have generally been used to deliver drugs to the posterior segment of the eye. These include delivery by the topical, systemic, intravitreal and periocular routes [11].

Non-invasive methods such as topical delivery are typically limited by static anterior segment barriers including the corneal epithelium, conjunctivae, sclera and choriocapillaris, and also, dynamic barriers such as lacrimation, blinking and conjunctival hyperaemia—these mechanisms resulting in lower drug bioavailability in the posterior segment tissues [12]. Gerard and colleagues [13] reviewed a gamut of approaches to the topical administration of macromolecules in preclinical models. The results suggested that the success of clinical study for posterior segment of the eye disease treatment through the topical route is still elusive. For example, factors such as the choice of model animals with similar physiology and anatomy with human and appropriate experimental design play an indispensable role in the feasibility assessment and the success of clinical translation of a therapeutic opportunity from animals to humans.

Theoretically, systemic application of drug administered via intravenous and oral route can also deliver to the posterior segment of the eye via the bloodstream. Nevertheless, drug entry is limited by blood-retinal barriers. The retinal epithelium and capillary’s endothelium are ‘tight’ and contains efflux transporters reducing the drug molecule’s permeability into the vitreous cavity [14]. Delivery via the systemic route is also inefficient since only a limited proportion of the arterial supply per circulation time is obtained by the eye. This exposes the non-target tissues to high concentrations and may lead to side effects.

The direct intravitreal route, which is commonly employed to treat retinal diseases, has become a standard technique to deliver drug substances to the back of the eye [15, 16]. The chronic nature of such conditions and the efficient intravitreal clearance necessitates that the patients will require recurrent injections using 27 G or smaller needles (up to 33 G needles). While intravitreal injections are a proven way to deliver therapeutic agents to the eye, the procedure is highly invasive and can create several harmful side effects, including retinal detachment, vitreous haemorrhage, cataract, the elevation of ocular pressure and ocular toxicity [17].

A less invasive choice for posterior segment delivery utilises periocular or transscleral routes such as retrobulbar, peribulbar, subconjunctival, and intrascleral delivery [18]. The objective is to establish a depot to allow continued exposure. When a drug is administered by transscleral delivery, it encounters multiple tissues and boundaries: episclera, sclera, choriocapillaris, Bruch’s membrane and the retinal pigment epithelium (RPE). There is the process which transport material away from the intended site of action or attenuate the action: blood flow, efflux pump, lymphatic drainage and a metabolic barrier, including lysosomal enzyme components and oxidation by cytochrome P450 isozymes protecting the neuroretina [19]. The drug bioavailability can be considerably reduced and lead to high dose requirement [20].

## Intraocular implants for posterior segment delivery

Intraocular implants, which are available in the form of biodegradable and non-biodegradable devices, are primarily developed to provide localised controlled drug release over a longer period of time. These devices help to bypass frequent intraocular injections, thus preventing related side effects. Biodegradable implants are generally fabricated using pharmaceutically acceptable polymers such as polylactic acid (PLA), polycaprolactones (PCLs), polylactic-co-glycolic acid (PLGA) and polyglycolic acid (PGA) [21]. Non-biodegradable implants have been fabricated using polymers such as silicone composite, polyvinyl alcohol (PVA) and ethylene vinyl acetate (EVA).

**Fig. 2:**
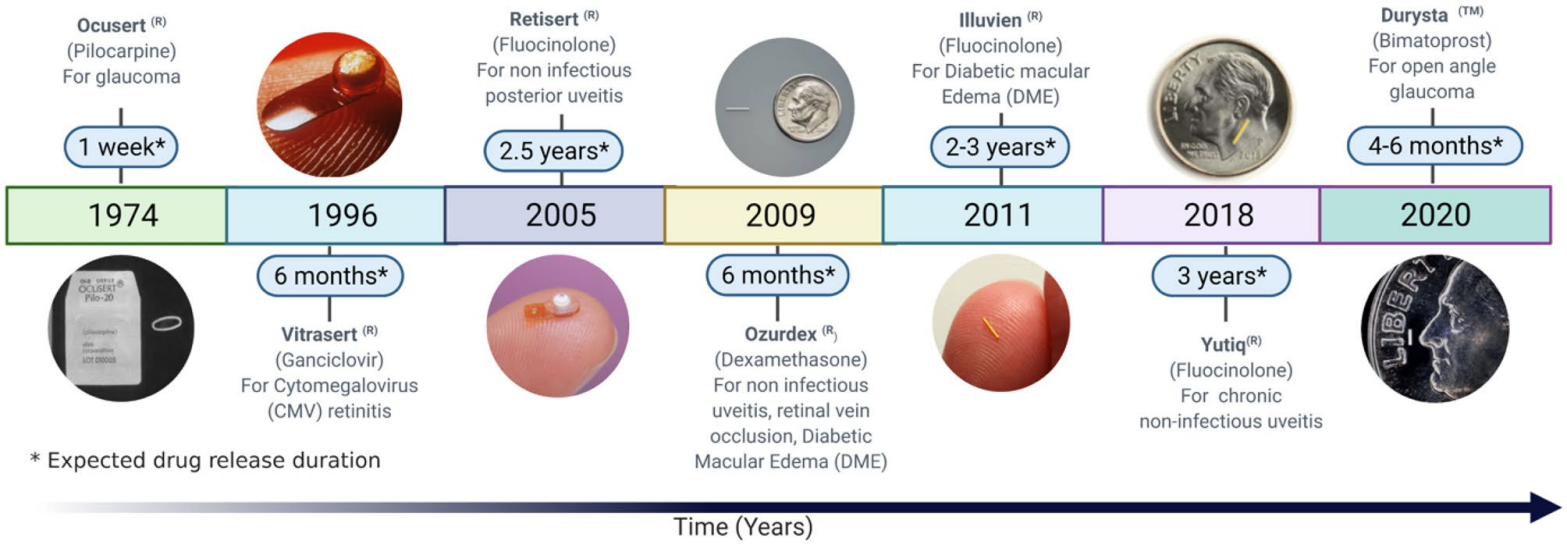
Development timeline for intraocular implants

The earliest attempts to provide sustained release were based on periocular devices placed under the lid. This included Ocusert®, which was the first FDA-approved ocular implant available in the market. It contained a reservoir of pilocarpine in a wafer between rate-controlling membranes and was engineered to deliver at drug release rates up to 50 μg h^−1^ [22]. Similarly, Lacrisert®, a hydroxypropyl cellulose rod that dissolves and thickens the tear film to treat dry eye, is placed in the conjunctival sac. This device was introduced in the early 1980s, but unlike Ocusert® is still marketed [23]. Over nearly 46 years, only seven intraocular implants have been successfully introduced into the market. More recently, a bimatoprost-loaded (Durysta™) containing 10-μg drug was approved by the FDA [24] (Fig. 2), while non-biodegradable implants containing fluocinolone acetonide (Retisert®, Iluvien®, Yutiq™) and ganciclovir (Vitrasert®) demonstrate reliable and zero-order kinetics release patterns. Surgery is needed to remove these implants post-drug release, leading to more invasive treatment than their biodegradable counterpart. Ozurdex®, which is intended for the continuous delivery of dexamethasone to treat macular oedema (ME) and intraocular inflammation, is an example of a biodegradable implant for posterior segment delivery [25]. Ozurdex® and Durysta™ employ the biodegradable NOVADUR® technology of Allergan for the delivery of dexamethasone and bimatoprost, respectively. A PLGA polymer matrix is used in the NOVADUR® method, which slowly degrades to lactic acid and glycolic acid, allowing for a sustained drug release for 6 months [26]. Table 1 summarises the marketed implantable ophthalmic drugs for posterior segment delivery. Table 2 illustrates several types of implants for the treatment of posterior segment eye disease at different stages of clinical phase investigations.

**Table 1:** Marketed Intravitreal implantable devices for posterior segment delivery

Name	Drug loaded	Target	Polymer type	Duration	Implant manufacturer	Status	Reference
Yutiq™	Fluocinolone acetonide (0.18 mg)	Posterior uveitis	Polyimide/PVA	3 years	EyePoint Pharmaceuticals, USA	FDA approval 2018. Marketed in the USA	[36]
Illuvien®	Fluocinolone acetonide (190 μg)	DME	Polyimide/PVA	2–3 years	Alimera Sciences, Inc., USA	FDA approval 2011 Marketed in the UK and Europe	[37, 38]
Ozurdex®	Dexamethasone (0.7 mg)	CRVO BRVO posterior uveitis	PLGA	6 months	Allergan, USA	FDA approval 2009 Marketed in the UK, Europe and USA	[39, 40]
Retisert®	Fluocinolone acetonide (0.59 mg)	Posterior uveitis, DME, CRVO	Silicone/PVA	2.5 years	Bausch & Lomb, Rochester, USA	FDA approval 2005 Marketed in the USA	[41]
Vitrasert®	Ganciclovir (4.5 mg)	CMV retinitis	EVA/PVA	5–8 months	Bausch & Lomb, Rochester, USA	FDA approval 1996 Discontinued (2013)	[42]

*CMV retinitis* Cytomegalovirus retinitis, *DME* diabetic macular oedema, *CRVO* central retinal vein occlusion, *BRVO* branch retinal vein occlusion

**Table 2:** Implantable intravitreal devices for posterior segment delivery under clinical investigation

Name	Drug loaded	Target	Implant type	Implant manufacturer	Status	Reference
IBI-20089 Verisome™	Triamcinolone	CME	Sustained-release intravitreal lipid–based DDS	Icon Bioscience	Phase II	[43]
I-vation®	Triamcinolone acetonide	DME	Titanium helical coil coated with TA	SurModics	Terminated	[44]
Tethadur	Proteins, antibodies and peptides	–	Biosilicon biodegradable	pSivida Corp	–	[44]
NT-503 (ECT)	Anti-VEGF drug molecules	Wet-AMD	Biodegradable implant	Neurotech Pharma	Terminated (2016)	[45]
Renexus	Ciliary neurotrophic factor (CNTF)	Macular telangiectasia type 2	Semipermeable hollow fibre membrane	Renexus Group & Noah Group	Phase III (2019)	[46]
ODTx	–	–	Laser-activated injectable rod implant	On Demand Therapeutics, Inc	–	[45]
NCT02087085	Brimonidine	AMD	Intravitreal implant	Allergan Inc	Phase II (2018)	[47]
NCT04060758	Latanoprost	Glaucoma	Latanoprost sustained release	PolyActiva Pty Ltd	Phase I (2020)	[48]
ForSight VISION4	Ranibizumab	AMD	Refillable port drug delivery system (PDS)	Genentech/Roche	Phase I	[49]

*CME* Cystoid macular oedema, *DME* diabetic macular oedema, *AMD* age-related macular degeneration

Recent biomedical engineering and ocular research have also promoted the development of sustained-release intraocular implants as investigational tools in treating ocular disease via periocular and intravitreal routes. For instance, Robinson and colleagues [27] described a PVA-based episcleral/intravitreal sustained-release implant to deliver gadolinium-pentaacetic acid (Gd-DTPA). The concentration of the radiopharmaceutical released from the intravitreal implant in the vitreous humour was 30 times higher than the episcleral implant. Okabe and colleagues [28] formulated a betamethasone phosphate–loaded scleral implant using a PLA polymer matrix for posterior segment delivery, and Fialho and colleagues [29] investigated the potency of poly(e-caprolactone) (PCL)-based intravitreal implant to deliver dexamethasone. Results showed that implants were able to release 25% of the drug in 21 weeks. McAvoy and colleagues [30] formulated triamcinolone and ovalbumin-loaded photocrosslinked intravitreal implants, showing that this implant could deliver drug over two months period.

Direct tapping into the eye to reduce pressure in refractory glaucoma in drainage surgery with the implantation of a bleed device has moderate success [31]. This approach seeks to reduce or avoid medications. The progress in the surgical implantation of devices, with good control of post-operative infection and high biocompatibility of the polymers, allows the implantation of devices such as the osmotic pump. Michelson and Nozik [32] describe an implantable osmotic mini-pump connected to the vitreous cavity of a rabbit. This device was able to deliver gentamicin over 4-day period. More recently, the construction of a reservoir that can be refilled with a biologic such as in the port delivery system [33] has shown considerable success.

Although these examples show the clear advantage of intravitreal delivery, less invasive routes are still sought. A Korean group [34] described delivery of triamcinolone acetonide through the transscleral route using a biodegradable intrascleral implant coated on one side and composed of PLA (poly(d,l-lactide). Directional coatings are used to reduce loss to the surrounding tissue and maintain higher concentrations at the primary tissue interface. Carcaboso and colleagues [35] attempted to formulate an episcleral implant to deliver topotecan, a topoisomerase-1 inhibitor, to the posterior segment of the eye. All these developments required appropriate in vitro/in vivo dissolution tests to optimise the dosage form and standardise production.

## Drug dissolution methods for implantable devices

In vitro dissolution testing in the pharmaceutical industry has become vital for optimizing dosage forms, including diffusion, deposition and dissolution of drug preparations as a monitoring and quality control tool in the manufacturing process [50, 51]. Dissolution testing can often assist in predicting the in vivo performance of a formulation and plays a significant role in bioequivalence (BE) studies related to product scale-up [52]. The choice of apparatus used when conducting the in vitro dissolution testing of dosage forms depends on several factors such as formulation, manufacturing process, drugs characteristics such as solubility and diffusion, method/apparatus used in the assay, dissolution medium and route of administration [53]. Factors such as pH, movement at the site, ionic strength, presence of enzymes and proteins, tissue structure and anatomical barrier must be considered when predicting the in vivo performance of drug formulated into ocular implantable devices. The scope for all these complex interactions cannot be reproduced easily in a simple in vitro dissolution setup. Since there are no compendial methods for in vitro dissolution testing of implantable ocular devices, experimental approaches related to in vitro dissolution testing of ocular implants have been adopted in various research labs. Ideally, these models must be able to demonstrate in vitro/in vivo correlation (IVIVC) and/or at least partially simulate the conditions of an ocular compartment for quick screening of potential formulations [54]. But due to the complexity of ocular physiology, barriers and site of implant administration, the concept of establishing IVIVC becomes challenging, therefore, appropriate in vitro tests must be investigated to simulate in vivo conditions. To address this a range of in vitro setups have been established, which are composed of a mix of either static or subject to light agitation methods are commonly employed for intraocular implant dissolution testing [55]. These includes modified compendial methods such as USP apparatus 3 (reciprocating cylinder), USP apparatus 4 (flow-through cell) and agar diffusion. However, recently, a number of customised in vitro setups have been investigated which are also discussed in the following sections in more detail.

### Static diffusion methods

**Fig. 3:**
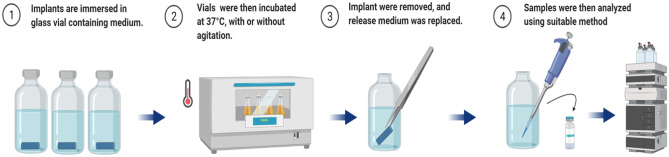
Schematic representation of a static dissolution setup (created with BioRender.com)

The static method is a straightforward method for determining the in vitro release of therapeutic agents from the implantable system (Fig. 3). In static methods, implants are placed directly into the appropriate release medium, which maintained at a constant temperature with or without agitation. Drug release is examined at predetermined time points by manually removing the implant from the release media or simply collecting the media itself. The concentration of drug in the supernatant is quantified using an appropriate analytical method [56]. Balasubramaniam and colleagues [57] compared different static and other dissolution methods to study the release of indomethacin from HPMC and sodium alginate implants. They concluded that the static method was not suitable for evaluating drug release from a compressed implant due to irregular swelling during the study. In this study, protein dissolution medium has been compared to porcine vitreous humour as the release medium, using static, semistatic and dynamic methods [58]. In the static method, precipitation, aggregation and protein instability issues were observed due to the inability of a static method to mimic the actual vitreous environment.

The static diffusion approach has been widely used to quantify the release of different drugs from in situ depot forming implants [56, 59, 60]. The drug-loaded polymers were directly injected into glass vials containing the release medium. The glass vials were then incubated at 37 °C, and sampling was taken at fixed intervals to quantify the amount of drug released. Static diffusion methods also have been incorporated to quantify the release of different drugs from solid implants [27–30, 34, 65]. Jiang and colleagues [61] investigated the release of bevacizumab from chitosan and polycaprolactone (PCL) electrospun fibres as a bilayered capsule, and the protein release from the capsule was carried out using a static dissolution assay. All the researchers used phosphate buffer pH 7.4 as media but with different volumes of the dissolution media, depending on the sink condition of each drug. Bode and colleagues concluded that the volume effect was negligible in their studies [56]. The incorporation of bevacizumab (Avastin®) into a novel intraocular device (capsule drug ring/CDR) provides a refillable reservoir for sustained drug release [62]. The authors used a static diffusion method based on 4-mL vials filled with balanced salt solution (BSS) media. Vials were placed in a heating pad, and 1-mL samples were collected sequentially. These methods were designed to control the influence of variables such as sink condition, agitation and temperature. However, static models cannot control the effect of the diffusion layer and ocular flows [63]. Researchers tend to adopt this method due to its availability, the straightforward and robust approach to determine drug concentration and sampling reproducibility [64].

### Agar diffusion methods

The advantage of the agar diffusion method is that it is already widely employed to examine the antimicrobial activity of drugs, for example against *Enterococci* [65]. It also can be developed to measure drug release in a high viscosity environment. However, this process does not mimic actual conditions because implants are designed to be placed in the hollow cavity surrounded by static gel substances, not by tissue and extracellular fluids represented in the ocular cavity [63]. In this method, the drug release from the implant is only controlled by a diffusion mechanism negating other factors that might reflect in the actual vitreous environment.

**Fig. 4:**
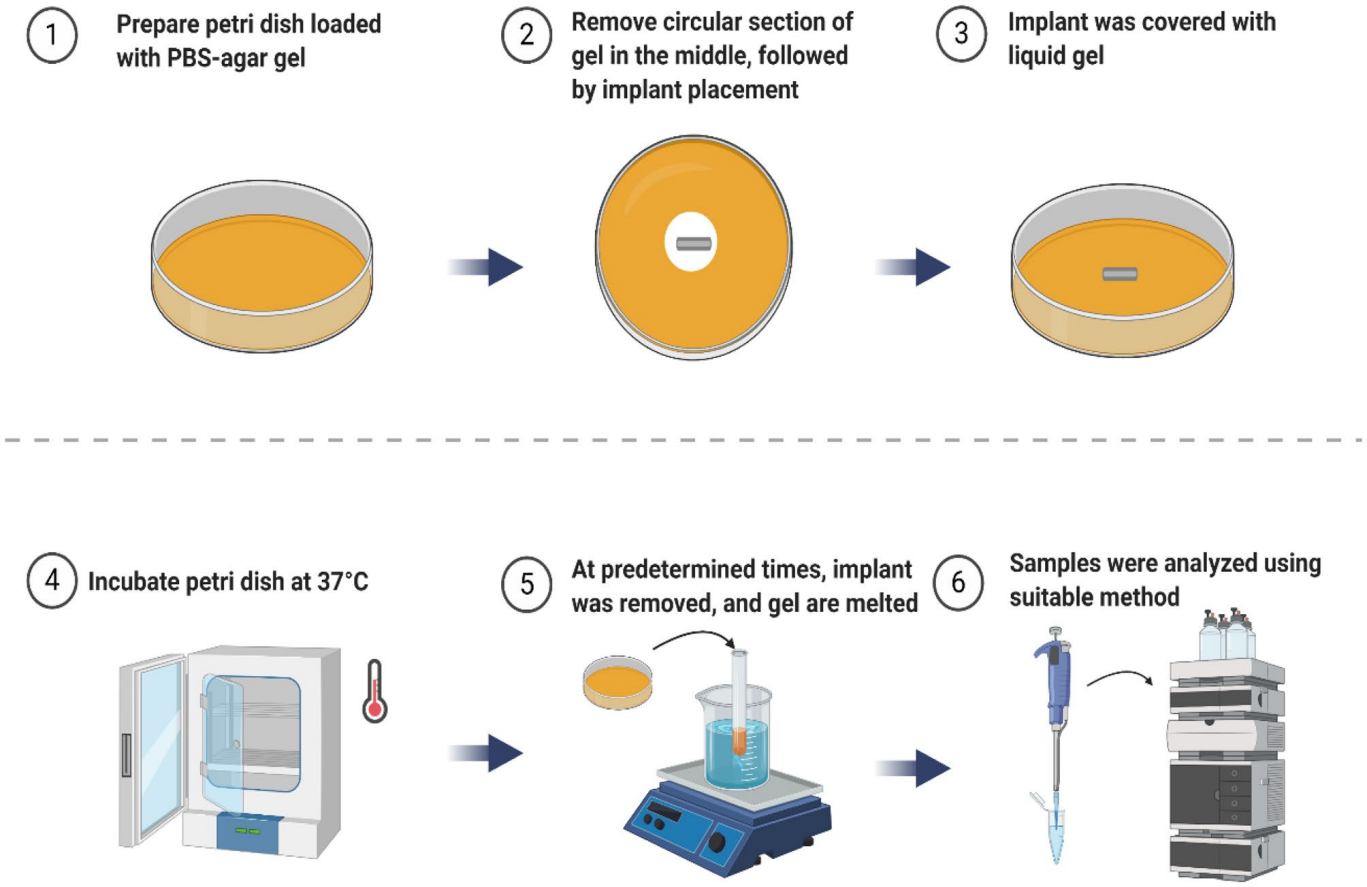
Schematic representation of agar diffusion setup (created with BioRender.com)

In the agar diffusion method, sterilised polysaccharide (agar) or protein (collagen) solution was poured into a petri dish to form a gel. Next, a circular section of gel in the middle of the plate was discarded and followed by the placement of the implant inside the hole and covered by liquid agar/collagen solution until it is solidified. At a predetermined time interval, implants were collected, and the gel is analysed using a suitable method to calculate the diffused drug in the gel (Fig. 4).

Balasubramaniam and colleagues [57] investigated the release of indomethacin from the implant using 1% and 2% w/v sterilised agar solution in the petri dish. The implant was placed in the centre of the petri dish and covered with an agar plug. The solidified agar was dissolved in hot phosphate buffer pH 7.4 to analyse the released indomethacin from the implant. Allababidi and Shah [63] used a similar method to study the release of cefazolin from glyceryl monostearate–based implants. The petri dish was divided into four zones, and samples were collected from each zone at defined time points. The release profile of cefazolin was compared with the static method using 0.1 M phosphate buffer pH 7.4 at 60 oscillation/min agitation. Results have shown that the agar diffusion method and the static method do not differ significantly. However, whereas the concentration of cefazolin in the static method was homogenous due to constant agitation, in the agar diffusion method, the released cefazolin diffused non-homogenously through gel establishing a concentration gradient.

### USP-based dissolution methods

There is no agreed and harmonised method for in vitro dissolution testing of long-acting intraocular implants. Although there is no specific USP dissolution apparatus qualified to simulate the physiological states, biochemical milieu or anatomical barriers present in and around the eye, USP apparatus 3, 4 and 7 have been investigated for in vitro studies of intraocular implants.

**Fig. 5:**
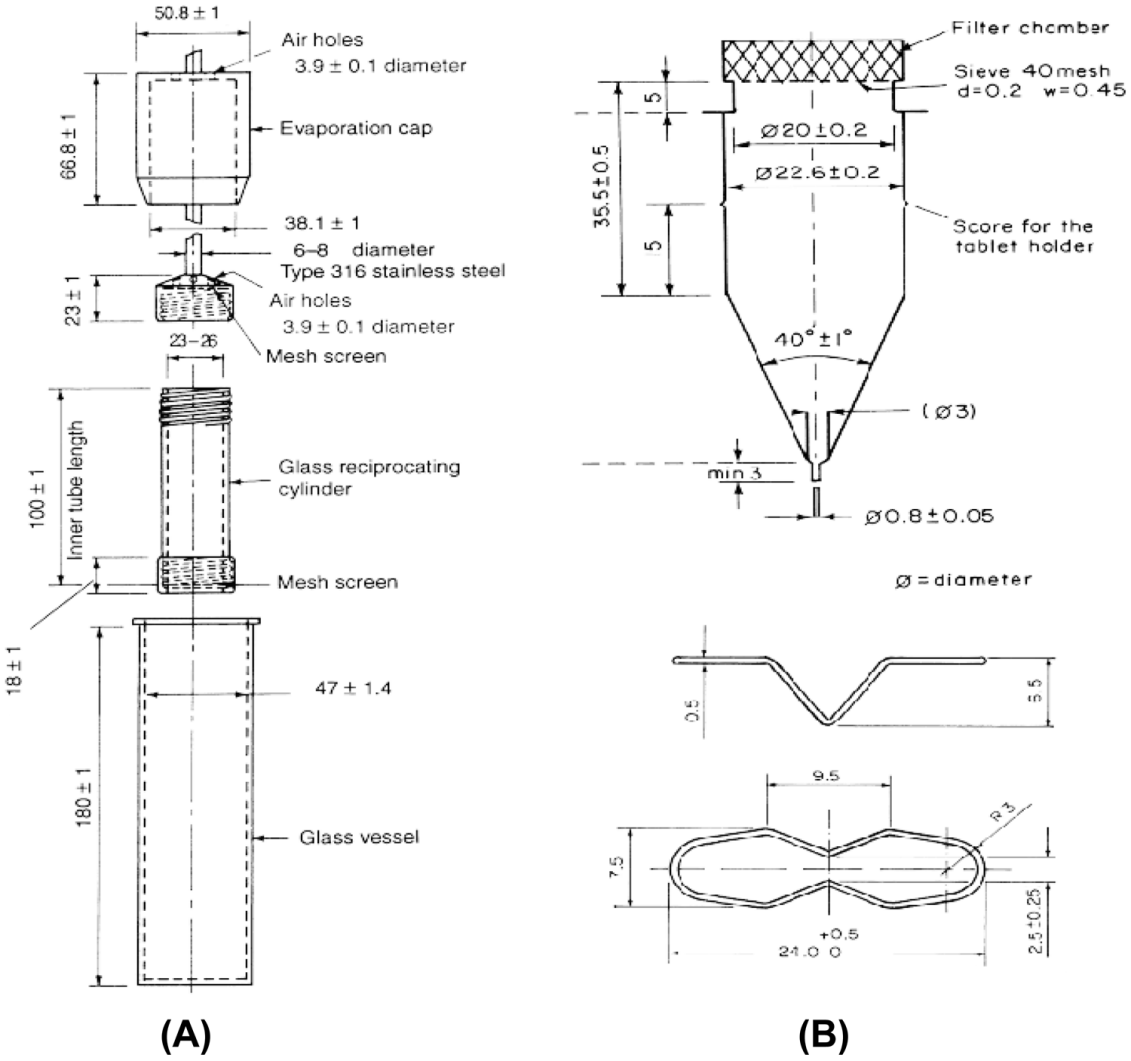
Schematics of USP apparatus 3 (**A**) USP apparatus 4 (**B**). Reproduced with permission from [73]

USP apparatus 3 (reciprocating cylinder) (Fig. 5A), which is commonly used in solid oral modified-release dosage form dissolution studies [66], has been used to investigate the release of ciprofloxacin from PLA/PLGA implant with a low volume of release media protected from evaporation during the dissolution study [67]. Samples were placed in the cylinder holder, and at a predefined time interval, 30 μL of the sample was collected from the dissolution medium and injected into the HPLC for quantification of ciprofloxacin. The data suggested that the drug release kinetics of the USP apparatus 3 which was comparable with the microdialysis system. Apart from describing the drug release mechanism through diffusion, this method also reported implant swelling behaviour that commonly occurs in biodegradable implants.

Among the different schemes of dissolution equipment available, the flow-through cell (USP apparatus 4) dissolution apparatus (Fig. 5B) has received much attention because of its flexibility and ability to study the dissolution of poorly water-soluble drug [68]. This technique also showed the reproducible and robust results, which is crucial for dissolution testing [69]. In addition, USP apparatus 4 can also be operated on low-volume release media and adjusted hydrodynamic flow conditions [70]. Shen and Burgess [71] reported using the USP apparatus 4 to study the dissolution of dexamethasone-loaded PLGA microsphere in PVA hydrogel implant composite. The samples were placed into the implant cells and media containing PBS pH 7.4 supplemented with 0.1% sodium azide to prevent bacterial contamination was circulated in the closed-loop system with an 8-mL/min flow rate. Rather than connecting the sample tube to the HPLC injector, 1-mL samples were collected at specified intervals for later HPLC analysis.

**Fig. 6:**
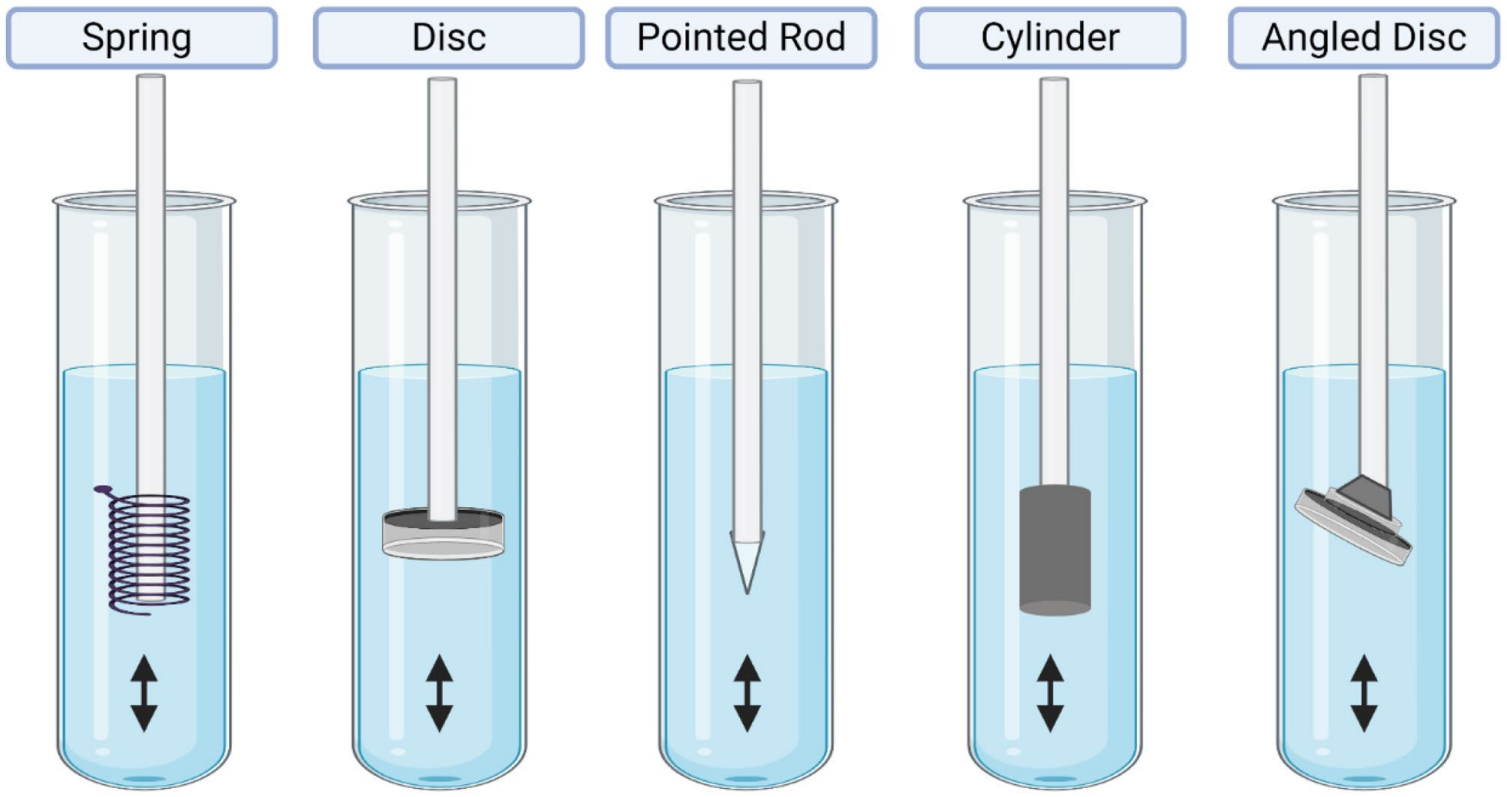
USP Apparatus 7, reciprocating holder (created with BioRender.com)

Stein and colleagues [72] proposed the use of USP apparatus 7 to study the in vitro release of a dexamethasone-loaded PLGA implant (Fig. 6). In this method, the sample cell was filled with Ringer’s solution pH 7.4. Next, the dissolution cells were heated to 37 °C and fitted in a mesh basket holder with 12-perforated openings, reciprocating at 20 dips per minute. The sample volume was set to 3.5 mL to simulate human vitreous humour with predetermined sampling time points; the whole medium was collected and replaced with a new fresh medium. Results showed that dexamethasone release displayed large variability due to occasional burst release. This unusual release pattern is triggered by the partial closure of the perforated base plate by implant fragments that obstructed the lateral metal struts of the reciprocal holder. Compared with other USP methods, USP apparatus 7 can simulate drug release not only through diffusion and swelling mechanism but also erosion pathway.

### Modified USP apparatus

Choonara and colleagues [74] used modified closed-compartment USP XXV dissolution testing apparatus to study the release of antiviral ganciclovir from novel doughnut-shaped minitablet. Samples were immersed in simulate vitreous humour (4-ml PBS with 0.03% hyaluronic acid, pH 7.4, 37 °C) then placed in the oscillating incubator 50 rpm. Results showed that this PLGA-based intraocular device degraded over time in the release medium, and a pattern of biphasic release was observed. Unfortunately, this article does not explain modifications in detail that was made using the USP XXV dissolution apparatus. Consequently, it is difficult to evaluate the advantages and disadvantages of the in vitro test method used in this publication. It is imperative to provide details of any modification to understand the scientific merits of these methods.

### Membrane system

The barriers that drug molecules meet before accessing the target location in the eye depend on where the implant is administered. For instance, in transscleral administration, the primary barrier is the sclera which composed of an interconnected matrix of collagen fibrils impregnated with ground substance. RPE also serves as the main barrier for periocular administration, limiting the entry of drugs from choroidal blood circulation [75]. Therefore, the incorporation of membranes in the in vitro dissolution apparatus might simulate the presence of these barriers, improving correlations with in vivo studies. Numerous attempts to simulate posterior segment eye barrier using different membranes have been studied using different approaches as follows:

#### Microdialysis system

**Fig. 7:**
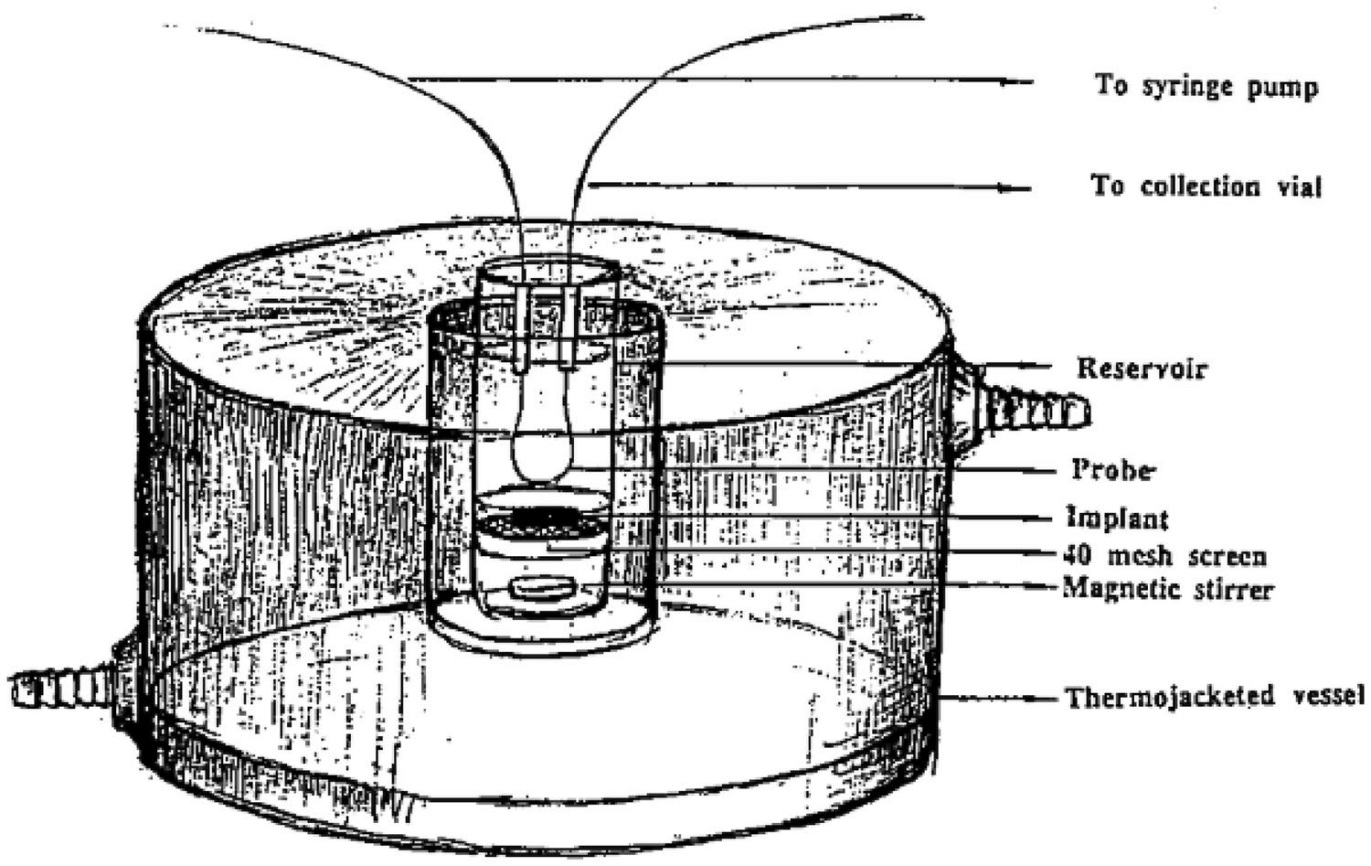
Microdialysis membrane developed by Dash and colleagues. Reproduced with permission from [70]

A technique to analyse drug release from implants by using a microdialysis system has been described by Dash and colleagues [70] (Fig. 7). Ciprofloxacin in a PLA/PLGA microcapsule compressed implant was placed in a 40-mesh screen inside Sorensen’s phosphate buffer (pH 7.4) medium reservoir. The medium was agitated at 50 rpm, and the flow rate was set to 0.5 to 1 μL/min. The perfusate samples were collected at a time interval and analysed using the HPLC method. Regenerated cellulose microdialysis hollow fibres 1300 Da was chosen as the dialysis membrane. This approach has benefits because it can continually monitor the drug over a period of time and be used to assess the concentration of the drug at the implantable site.

#### Dialysis bag

The use of dialysis membranes to investigate the in vitro dissolution of dexamethasone from bioerodible implant composed of microparticle PLGA and HPMC was described by Srinivas and colleagues [76]. A microparticle implant was fabricated by compressing microparticles until a pellet was formed. The implant was placed in a dialysis bag with 10 kDa MWCO (molecular weight cutoff) containing 1.0-mL balanced salt solution (BSS) pH 7.4. The bag was sealed at both ends and placed in a 100.0 mL of BSS pH 7.4 drug release medium followed by continuous stirring at 37 °C under the protection from light. Samples were collected at fixed time intervals and analysed by LC/MS/MS method. The result has shown that 50% of the drug was released over 22 days.

#### Modified Transwell cell

**Fig. 8:**
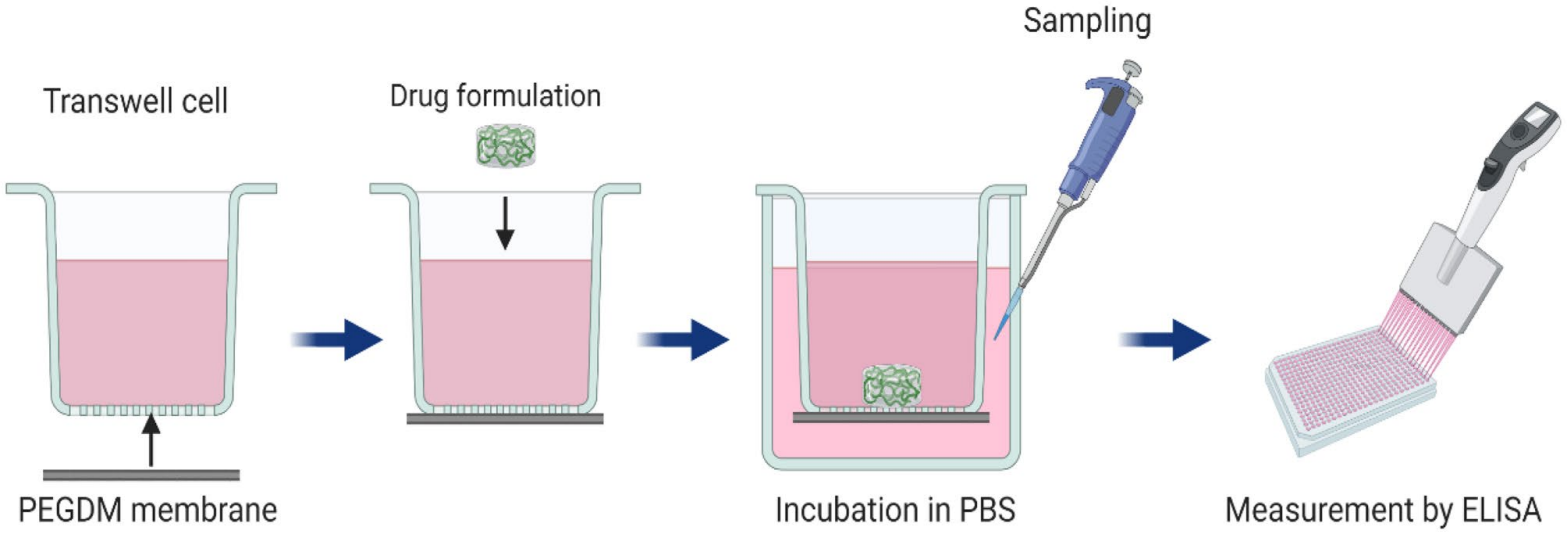
Schematic diagram of modified Transwell devices developed by Kawashima and colleagues. Reproduced with permission from [77]

A transscleral device consisting of a chamber made from moulded triethylene glycol dimethacrylate (TEGDM) and capped with a photopolymerised mixture of collagen (COL) and polyethylene glycol dimethacrylate (PEGDM) has been used for the development of brain-derived neurotrophic factor (BDNF), with prior development using 40-kDa fluorescein isothiocyanate dextran (FD40) to quantify membrane characteristics [77]. The membrane was permeable to molecules with molecular weights of < 200 kDa. Essentially, this research used a Transwell® device (Fig. 8) with the original membranes replaced with PEGDM/COL membranes. Capsules were each filled with BDNF-loaded collagen beads in PBS and sealed with membranes in which the concentration of COL was varied. Next, capsules were incubated in 1 mL of PBS at 37 °C. The results showed that by adjusting the amount of COL in the PEGDM/COL membrane, the drug release rate could be regulated.

### PK eye model

**Fig. 9:**
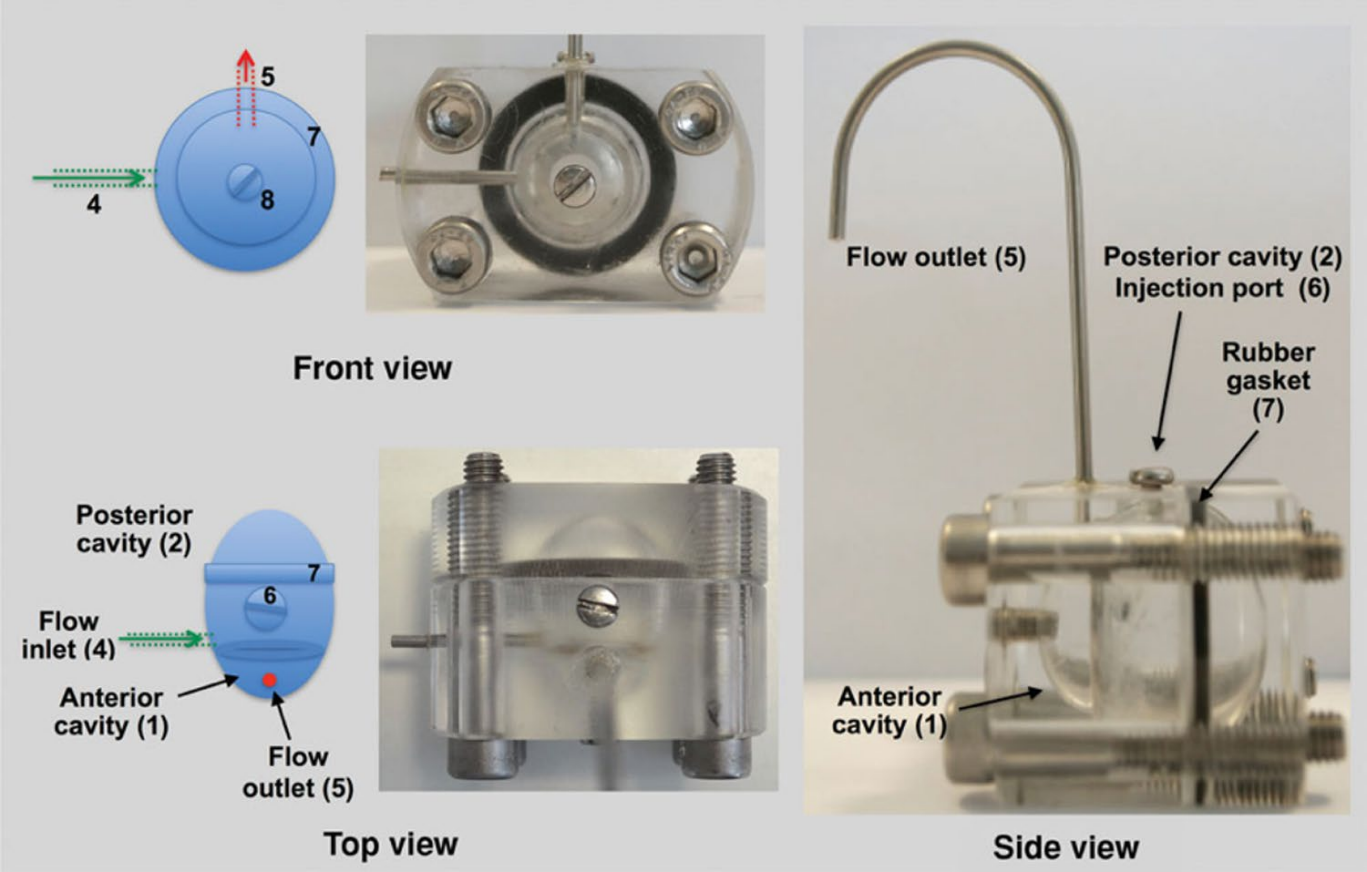
Top, front and side images of the PK-Eye model used in Sahar studies. Reproduced with permission from [78]

An innovative in vitro two-compartment model that enables an intraocular aqueous outflow (Fig. 9) was created to address and evaluate the clearance of drugs through the anterior segment [78]. It is proposed that this model can also be used to calculate the release of drug molecules from longer-acting ophthalmic therapeutic formulations located in the vitreous cavity. The device comprises two compartments separated by a membrane barrier composed of a dialysis membrane (12-14 kDa MWCO) simulating posterior and anterior cavities of the human eye. A mixture of PBS and simulated VH made from agar and hyaluronic acid (HA) with a dynamic viscosity of 0.6 Pa.s was used to resemble VH (0.5 Pa.s) in the posterior compartment [79]. The aqueous inlet port and the injection port were placed in the vitreous cavity. A sole outlet port was placed in the anterior chamber to simulate the outflow. This model was used to measure the dwelling times for ranibizumab, bevacizumab and triamcinolone acetonide (TA) suspensions. Results suggested that the PK eye model was able to demonstrate relevant clearance profiles between proteins (bevacizumab and ranibizumab) and poorly soluble drugs such as TA injected as a suspension, or potentially formulated as intravitreal implants.

### Eye movement system model

**Fig. 10:**
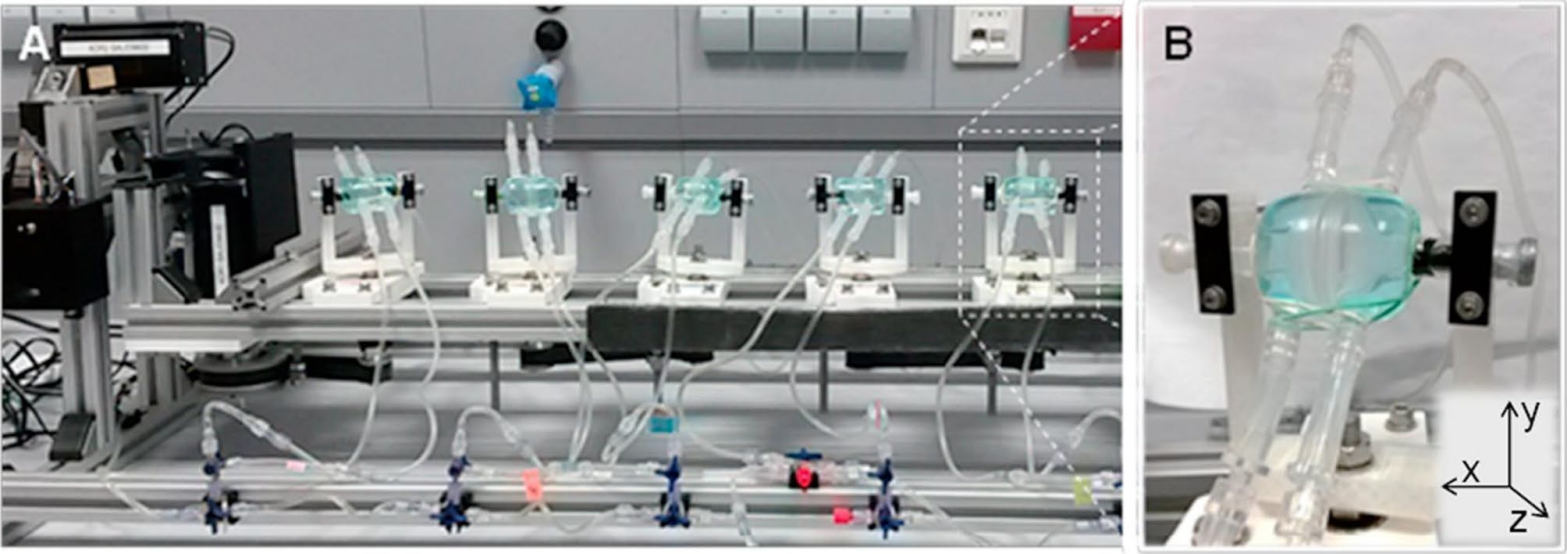
Eye movement system (**A**); vitreous models (**B**) developed by Stein and colleagues. Reproduced with permission from [72]

Within the eye, the translocation of materials from a depot results from diffusive and convective forces or, more correctly, advection as particle movement occurs in the fluid flow [80]. Therefore, oscillatory forces more correctly replicate the saccadic movements of the eyes and intentional movement of the head. An in vitro test (Fig. 10) has been described that combined a vitreous model (VM) [81] and a simple system described by Loch and colleagues [82] in an attempt to create an in vitro system resembling the vitreous body and the applied forces that move the depot [72]. A dexamethasone-loaded PCL-based implant was investigated in this experiment and loaded into VM filled with polyacrylamide gel (PAAG)-Ringer’s buffered saline as a release media which simulate vitreous humour. PAAG was used because it has a gel structure similar to young vitreous humour, while the Ringer’s buffered saline simulated the liquefied area (syneresis) commonly presents in the elderly [83].

On the eye movement system model (EyeMoS) setup, six VMs were configured in a horizontal row, and eye movements are simulated at a time interval of 24 h. The device was disassembled to remove the implant and the release medium at sampling times, and the implant was reinjected into a new medium within a VMM. Although the result exhibits an insignificant difference between the EyeMoS setup and a static method over the same period, the shape and dimensions of the implants remained stable when subjected with the EyeMoS setup. Furthermore, the effect of a gelled compartment and vitreous body liquefaction on the drug release can be analysed using this model.

### ExVit dynamic system

**Fig. 11:**
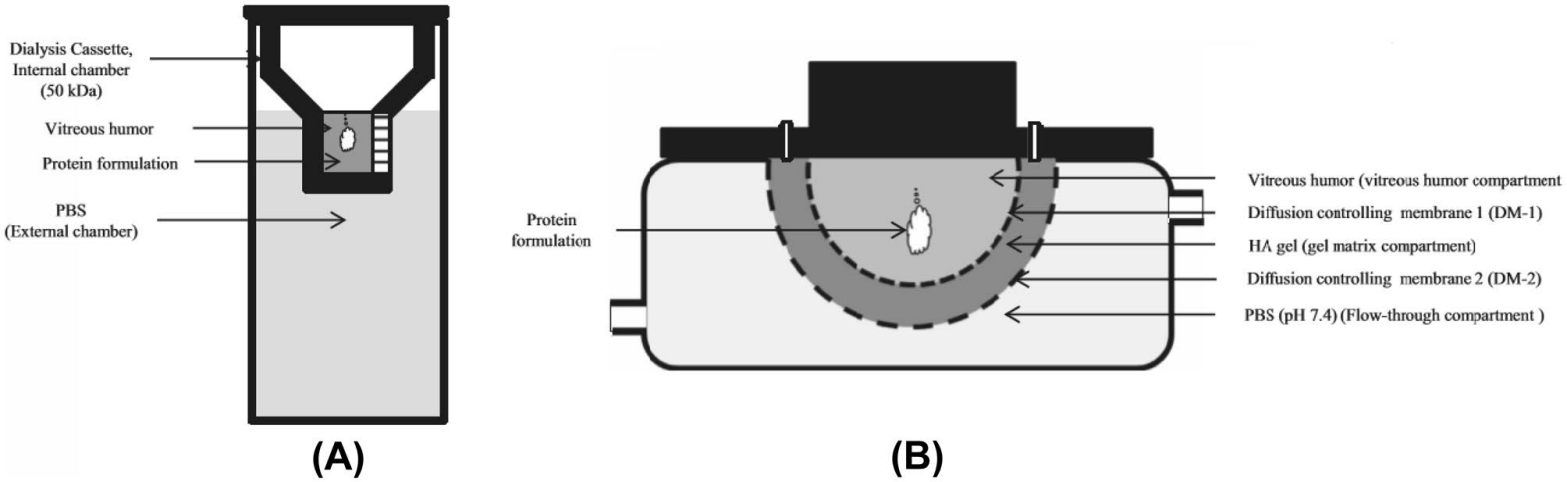
ExVit semidynamic (**A**) and ExVit dynamic (**B**) models developed by Patel and colleagues. Reproduced with permission from [58]

Patel and colleagues [58] developed two models, ExVit semidynamic and ExVit dynamic (Fig. 11), to examine the protein stability inside VH. Although these models were not intended for the dissolution study of an ocular implant, the design can be applied since this model incorporates a two-compartment system. The ExVit semidynamic model is composed of an inner compartment (filled with porcine VH) and an external compartment (filled with 0.01 M PBS). A 50 kDa MWCO membrane is used to insulate the compartments to simulate the literature values for the retinal exclusion limit [84]. The tools then sealed and incubated at 37 °C for 24 h. Samples from both compartments were taken and analysed using a suitable procedure.

The ExVit dynamic model contains three compartments. These are a vitreous compartment filled with porcine VH (PV), a gel-matrix compartment (GM) filled with hyaluronic acid gel in PBS, and a flow-through compartment (FT) loaded with PBS pH 7.4. GM acts as a diffusion limiting barrier, has separated PV and FT into two different compartments. The devices were secured and incubated at 37 °C for 24 h. Collected samples from all compartments then analysed using an appropriate procedure.

These two models were able to modulate the dissemination of different proteins, i.e., bovine serum albumin (BSA) and IgG, by adjusting the MWCO of the dialysis membrane. However, the authors concluded that this model was still insufficient to contemplate in vivo conditions and could not evaluate the pharmacokinetics of the intravitreal system.

## Dissolution medium in the in vitro setup

It is accepted that many parameters affect drug release from a formulation, including the dissolution medium, temperature, pH, agitation rate and presence of enzymes mechanism [85]. The mechanisms involved and the magnitude of the effects may be formulation and drug-specific. The medium composition used for drug release studies has a vital role in the success of dissolution studies. Ideally, the media must be produced according to ocular physiological conditions [54].

For posterior segment delivery systems, the conditions chosen are primarily adjusted to VH characteristics, a gel-like body that occupies considerable portion of the eye. The VH contains 98 to 99% water, salts, hyaluronic acid, proteins and collagen [86]. The VH volume depends on age, varies between 1.6 and 4.8 cm^3^ with the pH value around 7.0–7.5 and the density value of 1.0053–1.0089 g/cm^3^ [87, 88]. In newborns, the volume of VH is 1.6 mL with an all-gel consistency. With ageing, the VH changes from a gel-like substance to a fluid-like substance. The liquid vitreous occurs for the first time at the age of 10 and increases gradually until it occupies almost half of the vitreous at the age of 70 [87].

To simulate the VH conditions, the majority of drug release studies use phosphate buffer saline (PBS) pH 7.4 as a release medium [20–26, 48, 52, 55, 61, 62, 68]. Choonara and colleagues [74] incorporated hyaluronic acid in the media to maintain the viscosity of PBS to imitate the gel properties of the VH. Other studies use buffered media having similar pH and ionic strength to VH (7.0–7.4) [89], such as balanced salt solution [62, 76], Sorensen buffer [70] and Ringer’s buffered saline [72]. In addition to liquid media, viscous and gel-like media such as agar mixture [57, 63], polyacrylamide gel (PAA-gel) [72], were also used in the different setups. Porcine VH and PAA-gel have similar physical properties (water content, pH, viscosity, density and refractive index) to human VH [81]. Awwad and colleagues [78] prepared artificial VH that consists of a mixture of agar and hyaluronic acid to mimic the viscosity of human VH, and porcine VH has been used directly as the dissolution media [58]. Various in vitro drug dissolution studies of the ocular implants are shown in Table 3.

**Table 3:** Different conditions of in vitro dissolution setups of ocular implants

**Drug**	**Implant type and composition**	**In vitro dissolution method**		**Application mode of implant**	**Release media**	**Temperature/agitation speed**	**Reference**
		**Method**	**Membrane**				
Gadolinium DTPA (Gd-DTPA)	episcleral/intravitreal discs	Static	No membrane	Implant was submerged in the medium	PBS pH 7.4	37 °C, 150 rpm agitation	[27]
Triamcinolone acetonide	Solid episcleral/intravitreal	Static	No membrane	Implant was submerged in the medium	PBS pH 7.4	37 °C	[34]
Bethametason phosphate	Solid transscleral	Static	No membrane	Implant was submerged in the medium	PBS pH 7.4	37 °C, shaking	[28]
Dexamethasone	Intravitreal	Static	No membrane	Implant was submerged in the medium	BSS (balanced salt solution, pH 7.4)	37 °C, 30 rpm	[29]
Triamcinolone acetonide, ovalbumin	PEGDA-based intravitreal implant	Static	No membrane	Implant was submerged in the medium	PBS pH 7.4	37 °C, 30 rpm	[30]
Bevacizumab	Electrospun fibre bilayered capsule Intravitreal	Static	No membrane	Capsule was immersed in a tube containing medium	PBS	37 °C, no agitation	[61]
Bevacizumab	Intraocular capsule drug ring (CDR)	Static	No membrane	CDR was immersed in a tube containing medium	Balanced salt solution	37 °C	[62]
Buserelin acetate	Not defined	Static	No membrane	Implant was immersed in a glass vial containing medium	0.1% sodium azide and 0.05% benzalkonium chloride in phosphate buffer pH 7.4,	37 °C, no agitation. Samples were shaken 5 s at sampling time	[64]
Topotecan	Solid episcleral	Static	No membrane	Implant was submerged in the medium	PBS pH 7.4	37 °C, no agitation	[65]
Indomethacin	Solid intraocular	Agar diffusion	Agar gel as membrane	Implant was put in the middle of an agar gel-filled petri dish	1 and 2% w/v agar solution in water	Not defined	[57]
Cefazolin and ciprofloxacin	Glyceryl monostearate-based implant	Agar diffusion	Agar gel as membrane	Implant was put in the middle of an agar gel-filled petri dish	1.5% agar solution in 0.1 M phosphate buffer	37.5 °C	[63]
Indomethacin	HPMC-based intraocular	USP apparatus 1 dissolution	No membrane	Implant was placed in the basket which has 50 rpm rotation speed	PBS pH 7.4	37 °C	[57]
Monoclonal antibodies and fragment antibodies	Not defined	ExVit system	50MWCO membrane (semidynamic) Two diffusion membrane (dynamic)	Samples were injected into the vitreous compartment and incubated	Porcine vitreous humour and PBS	37 °C	[58]
Ciprofloxacin	PLA/PLGA-based implant	USP apparatus 3 dissolution	No membrane	Implant was placed in the cylinder holder	Sorensen’s buffer	37 °C	[70]
Not defined	Not defined	open loop-USP Apparatus 4 dissolution system	No membrane	Implant was mounted on glass beads in a 22.6-mm flow-through cell. The outlet hose of dissolution unit was connected to the HPLC injection valve	PBS pH 7.4	37 °C	[90]
Dexamethasone	PLGA /PVA hydrogel composite intravitreal implant	closed loop-USP Apparatus 4 dissolution system	No membrane	The samples were put into the implant cells and release media was circulated in the closed-loop system	PBS pH 7.4 with sodium azide 0.1%	37 °C	[71]
Dexamethasone	PLGA-based intraocular	USP apparatus 7 dissolution	No membrane	Implant was placed in the heated dissolution cells equipped with 3 or 12 opening basket, which reciprocate 20 dips per minute	Ringer buffer pH 7.4	37 °C	[72]
Dexamethasone	PCL-based intraocular	EyeMoS model	Not defined	Implants were inserted into VMs, and for 24 h, EyeMoS was set to a certain sequence. The VM was disassembled, implant and release media were collected at the given sampling times	polyacrylamide gel (PAAG)-Ringer buffer	37 °C	[72]
Gancyclovir	doughnut-shaped minitablet intraocular	Modified closed compartment USP apparatus	No membrane	Devices were immersed in media and oscillated	PBS with 0.03% hyaluronic acid	37 °C with 50 rpm oscillation speed	[74]
Ciprofloxacin	PLA/PLGA-based implant	Microdialysis membrane system	Regenerated cellulose microdialysis hollow fibres 1300 Da MWCO	Implant was placed in the 40 mesh screen inside the reservoir	Sorensen’s buffer	37 °C with 50 rpm constant stirring of medium	[70]
Dexamethasone	PLGA-based bioerodible ocular implant	Dialysis bag	10KDa MWCO	Implant was put in dialysis bag containing release media. The bag was closed at both end and placed in 100.0 mL release medium	BSS pH 7.4	37.0 °C, 70 rpm stirring	[76]
Brain-derived neurotrophic factor (BDNF)	Transscleral membrane–sealed capsules	Modified transwell cell	collagen microparticles membrane	Capsules were immersed in 1 mL of release media at 37 °C inside Transwell cell	PBS	37 °C	[77]
Triamcinolone acetonide, bevacizumab and ranibizumab	Intravitreal depot suspension	PK-eye model	12-14KDa MWCO	Via its injection port, PBS was applied to the anterior cavity. Drug was injected into the vitreal cavity and sampled from the outflow port	Artificial vitreous made from agar and hyaluronic acid	37 °C	[78]

## In vitro–in vivo correlation of ocular implantable devices

In vitro–in vivo correlation (IVIVC) is a mathematical methodology that explains the connection between in vitro drug dissolution within release media (drug release profiles) and in vivo performance (measurement of cumulative absorption from plasma concentration) of the same dosage form [53, 91]. US Food and Drug Administration (FDA) defines IVIVC as “a predictive mathematical model describing the relationship between an in vitro property of a dosage form and an in vivo response”. It has been graded into five distinct levels, including levels A, B, C, D and multiple levels C [92].

- Level A (in vitro dissolution vs in vivo absorption) depicts a linear relationship between in vitro and in vivo response. Level A correlation is known to be the most detailed and is recommended by FDA. It is also generally used to obtain biowaiver since it allows changes in material sources and manufacturing sites, as well as small improvements in the formulation.
- Level B (in vitro mean dissolution time vs in vivo mean absorption time) compares all the available in vitro and in vivo results using reference statistical moment analysis. However, level B is not a linear correlation and does not depict the accurate in vivo plasma profile.
- Level C (single point relationship) correlate between a dissolution parameter (e.g. T_50_) with in vivo parameters such as AUC, T_max_ or C_max_. The predictive in vivo pharmacokinetics of the drug formulation using level C correlation is limited because it does not represent the full plasma concentration–time plot [93].
- Multiple level C (interaction of one or more pharmacokinetic parameters) correlate between various dissolution time points spanning initial, middle and late dissolution periods with AUC, T_max_ or C_max_. This level can be as valuable as a correlation at level A.
- Level D (qualitative analysis) is a rank order correlation contrasting in vitro and in vivo release profiles. For regulatory purposes, it is not considered useful but may assist in producing a formulation [94].

These levels of IVIVC is well established for oral dosage form but limited progress with complex non-oral dosage forms such as ocular implant due to lack of in vitro testing method, their multi-phasic release and difficulty of deconvolution technique to establish the correlation. Therefore, only a few research attempts to correlate in vitro and in vivo release of ocular dosage form [64, 95, 96], which is not internally and externally validated according to the requirement of level A IVIVC. To the best of our knowledge, no regulatory IVIVC guidelines for the evaluation of ocular implantable devices are available so far. However, the same concepts of IVIVC development for oral extended-release dosage forms may apply [64, 95].

## Challenges of developing a suitable IVIVC for ocular implant

The most challenging features of conceiving an IVIVC for the long-acting ocular implant is to design an in vitro dissolution setup which reflects the in vivo behaviour as much as possible. However, imitating the drug distribution and permeation mechanism in the eye is more complicated than other routes — e.g., changes with ageing and disease condition such as the liquefaction of VH in age-related macular degeneration (AMD) have a high impact on product performance and interindividual variability. Furthermore, the in vitro setup should be appropriate for the complexity of the drug release mechanism and the time required for real-time in vivo testing. Tan and colleagues [97] tried to overcome this by developing a liquefied vitreous model, which considers vitreous liquefaction thus simulates the aged condition. This study investigated the distribution of fluorescent molecules using retinal angiography and ocular fluorophotometry. By using a non-invasive method, the drug concentration gradient across the vitreous chamber can be investigated. The limitation of this method is that the probe commonly used in fluorophotometry may be significantly chemically different from the drug of interest.

Another challenge is to get serial measurements of drug concentrations in a live eye since putting invasive serial probe sampling in the human eye is not feasible for ethical purposes. This aspect is essential to achieve level A IVIVC regulatory related to drug approval or determine drug dose regimens based on pharmacokinetic parameters [98]. The researcher usually constructs time curves from multiple studies since only single sample can be obtained at paracentesis in eye surgery. The best attempts have been made with the rabbit microdialysis model, allowing continuous monitoring of drug concentration in the eye tissue with minimal interference. To determine the ocular pharmacokinetics of ciprofloxacin eye drops, Klaus and colleagues [99] suggested surgically inserted microdialysis perfused probes in the anterior and vitreous segments of a rabbit model. Macha and colleagues [100] also use a similar approach to study pharmacokinetic parameters such as T_50_, elimination rate, clearance and mean residence time (MRT) of ganciclovir intravitreal injection.

Ocular implants are typically administered through direct injection into different areas (e.g., intravitreal, periocular), and drugs are released slowly from the biomaterial vehicle into the tissue fluids via swelling, diffusion, polymer degradation or combination thereof [101]. Next, drugs are transported into the target tissue via diffusion or other mechanisms. All the in vitro dissolution testing explained previously still have not reflected accurately the presence of multiple barriers inside ocular cavities [102]; the presence of multiple proteolytic enzymes and traces of creatinine, urea, xanthine and hypoxanthine inside VH [103]; presence of efflux pump and drug transport mechanism [104]; and other factors which influence the pharmacokinetics aspect of ocular drug delivery [105]. Several in vitro setups described previously, such as agar diffusion, microdialysis system, PK-Eye model and Ex-Vit system have been developed to address the presence of barrier membrane. Still, none of them can mimic the complexity of the eye. EyeMoS model was developed to address these challenges in the drug release mechanism, but further improvements are needed to overcome the other factors [106].

Ocular implants commonly show biphasic or even multiple phasic release profiles [107]. This is largely dependent upon the type of biodegradable polymer used in the fabrication of the implants for, e.g., PLGA shows a triphasic drug release profiles, i.e., burst release, diffusion and biodegradation (bulk erosion). The burst release arises due to the release of the drugs on the surface of the implants, during the lag-phase of a depot forming implants or combination of both. In contrast, in the diffusion phase, the drugs release slowly and typically determined by drug loading, polymer (type, molecular weight) and implant composition/dimensions. To help establish IVIVC for ocular implants, different mathematical models that introduce parameters that characterise the degree of dissolution (e.g., Higuchi and Korsmeyer-Peppas) have been applied. Schliecker and colleagues [64] and Tamaddon and colleagues [108], for instance, attempted to create an IVIVC correlation on which the Higuchi model would explain the drug release. However, in vivo data can be challenging to compare with multiple phasic release data using a basic standalone statistical model. For example, it is hard to predict the initial release of a drug inside periocular implant formulation based on in vitro data due to the in vivo rate-limiting step that is determined by the drug penetration through sclera while in vitro is drug solubility in the polymer matrix [109]. Furthermore, pharmacokinetic parameters (AUC, T_max_ or C_max_) which are difficult to obtain in the long-acting intraocular implant due to precorneal fluid drainage, drug-protein binding in the VH, systemic absorption and drug metabolism are often needed in the deconvolution technique to establish an effective mathematical correlation [110].

Mathematical models which define relevant compartments permitting accurate estimation of distribution and clearance of intravitreally injected drugs also needed for IVIVC establishment of intravitreal implants. Several in vitro methods of various complexities, such as PK Eye, Ex-Vit system and EyeMoS model, also address this although a good correlation still hard to accomplish. A good approach to define relevant compartments is by developing in silico models for the eye using finite-element modelling, which is commonly applied in engineering to model physical phenomena in certain systems. Friedrich and Park [111] have used this method to predict drug distribution within the VH accurately. A similar model developed by Missel [112] was also able to predict drug clearance after intravitreal injections in one species based on experimental results obtained from another species.

Typically, an actual in vitro testing of intraocular implants takes a long time. Accelerated in vitro testing is therefore necessary for early screening if formulations. Ideally, real-time and accelerated in vitro drug release tests demonstrate a similar release mechanism with a linear association between profiles [113]. However, an accelerated test performed under extreme conditions (extreme pH and high temperature) has often been shown to promote a different drug release mechanism compared with real-time testing [85]. High temperatures can lead to drug instability (especially biologics) and increased polymer mobility, resulting in different drug release profile, while extreme pH can accelerate the degradation of specific polymers. A suitable accelerated test must be thoroughly evaluated to overcome this challenge.

## Future prospective

According to the FDA, the demonstration of bioequivalence for non-solution dosage forms is recognised to be challenging, especially for ophthalmic products that are locally acting. A comprehension of the relationship between physicochemical properties of ocular implants and their bioavailability is important in designing pharmaceutical bioequivalent implant. In 2016, there were only 8 reported projects focusing on the development of in vitro release models that could represent the in vivo performance [114]. At the present time, there does not appear to be a harmonised regulatory standard (FDA/EMA) for in vitro dissolution methods applicable to the assessment of ocular implantable devices that have gone beyond simple dissolution testing. This stimulates the exploration of a various customised experimental protocols with regard to tools design, dissolution medium, membrane selection, sampling time, temperature and agitation rate. With the increasing number of commercialization activities in the development of long-acting ocular implants, it is desirable to develop suitable IVIVCs to assure product safety and enhance product development and screening activities. At present, there is a paucity of information in this critical field. Pragmatically, it is easier to develop in vivo — pharmacodynamic assessment methodology as smaller numbers of animals are needed, and these data should be relatable to cell-based biological activity assays. Thus, future research should fully validate either improved versions of existing in vitro testing methods or assist in developing new methods that have a better correlation with in vivo measurements. In addition, improved computer simulations based on compartmental and finite element modelling are needed to assist the exploration of the in vitro/in vivo relationship for ophthalmic formulations.

## Data Availability

Not applicable.
